# The Therapeutic Value of Arsenauro1Read before the Mississippi Valley Medical Association, at Louisville, October 7, 1897.

**Published:** 1898-03

**Authors:** A. P. Buchman

**Affiliations:** Fort Wayne, Ind.


### Selection.

### THE THERAPEUTIC VALUE OF ARSENAURO.[1]

BY A. P. BUCHMAN, M. D., Fort Wayne, Ind.

TO FULLY appreciate the therapeutic value of a drug one must understand its limitations. No remedy can be made to do more than a limited number of things. To ascertain just what pathologic conditions are modified or changed for the better by a given therapeutic agent is a task of no mean importance, yet an absolute necessity when we aim to be rational in our methods.

For the past four years arsenauro has been one of the chief factors in my therapeutic armamentarium because of its almost universal happy effects in the special line of work that I have, almost exclusively, engaged in. The body of work has been in the field of denutrition and false metabolism depending remotely upon gastric and intestinal indigestion.

1. Read before the Mississippi Valley Medical Association, at Louisville, October 7, 1897.

It is not my purpose at this time to classify and enumerate an extensive list of such patients, but rather to give a very short clinical history of a type case in which the phenomena that reached the threshold of consciousness were sufficiently distinct to induce the opinion that a diverse etiology, rather than a single line of cleavage, was necessary in order to reach a logical demonstration of the causative factors in operation, and therefore rationally outline a therapeutic course destined to terminate in satisfactory results.

Abnormal metabolism and denutrition express themselves in direct relation with constitutional idiosyncrasies, hygienic environments and the vulnerability of the organism. Bearing this in mind we can comprehend why one patient will present a pathology of the lungs, another a kidney affection and still another a disease of the nervous system, while the point of departure from the health line in all is the same.

The first case in which I noticed gratifying results following the exhibition of arsenauro, was that of a traveling insurance adjuster who had suffered with gastric indigestion over a period of five years, in consequence of which his blood stream was impoverished, his nervous system shattered and the whole organism working at the lowest possible pressure. The particular symptom that brought him to me was insomnia. He was forced to quit work on this account. A further description of this case is unnecessary, as the clinical picture is familiar to everyone. A thorough cleansing and disinfection of the digestive tube was the first step, after which I carefully regulated the diet so as to insure the greatest quantity of nutrition for the least amount of energy expended by the digestive forces. Bathing, massage and electricity were ordered. The usual carminative and tonic drugs were exhibited. This course was persisted in for a month, during which there was noticeable betterment, but not sufficient to satisfy either the patient or myself. I now withdrew all former drugs and gave him arsenauro in ten drop doses four times daily. In ten days the patient was sleeping comfortably, eating and digesting fairly well, and altogether was sufficiently recovered to go to work moderately. After sixty days' constant use of the drug he announced himself as having entirely recovered and able to perform the exacting work required of him with ease and pleasure.

The recital of this case will suffice to illustrate the groove into which arsenauro fits so perfectly. It changes the chemical movement in the blood plasma. The movement of the atoms thus initiated continues ; new material takes a more pronounced part in the various phenomena of motion and life ; the lymphatic glands, whose office it is to supply fresh material to the blood and nervous system, are changed to healthy action, their products become normally reconstructive ; cell digestion is stimulated and the blood is improved up to a normal standard.

To accomplish these results, however, it is not enough to simply give arsenauro. I have tried to expose the preparatory work which is absolutely essential and without which arsenauro, like any other drug given out of time and place, will yield only negative or indifferent results.

I have never secured from Fowler's solution the fully desired arsenic results which have invariably followed the administration of arsenauro, and yet, as Dr. Stuckey over a year ago pointed out in his scientific paper, the average dose of arsenauro contains very much less actual metallic arsenic than does Fowler's solution. We evidently have an entirely new agent in arsenauro, something more than the mere combining of arsenic and gold, for by evaporating arsenauro you have a resultant crystal which is not the crystal of arsenic, nor is it the crystal of gold, but a crystal such as I have never seen before. I would lay emphasis upon the point that I have observed no evidence of arsenical poisoning from arsenauro. It does not produce cumulative effects, but is easily and promptly assimilated.

